# The role of dairy in healthy and sustainable food systems: community voices from India

**DOI:** 10.1186/s12889-022-13194-w

**Published:** 2022-04-22

**Authors:** Kerry Ann Brown, Nikhil Srinivasapura Venkateshmurthy, Gopi Potubariki, Piyu Sharma, Jacqueline M. Cardwell, Dorairaj Prabhakaran, Cecile Knai, Sailesh Mohan

**Affiliations:** 1 College of Life & Environmental Sciences, University of Exeter, Exeter, UK; 2 Faculty of Public Health & Policy, London School of Hygiene & Tropical Medicine, London, UK; 3 Centre for Chronic Disease Control, New Delhi, India; 4 Public Health Foundation of India, Gurugram, India; 5 Department of Pathobiology & Population Sciences, Royal Veterinary College, London, UK; 6 Department of Epidemiology, London School of Hygiene & Tropical Medicine, London, UK; 7 Faculty of Health, Deakin University, Burwood, Melbourne, Australia

**Keywords:** Photovoice, Participatory research, Dairy, Food systems, Sustainable development goals, India

## Abstract

**Background:**

Managing the role of dairy foods in healthy and sustainable food systems is challenging. Milk production is associated with greenhouse gas emissions and milk-based processed foods can be high in fat, sugar and salt; yet, milk production provides income generating opportunities for farmers and dairy foods provide essential nutrients to young children, with a cultural significance in many communities. This is particularly relevant to India, the world’s largest producer of milk. The aim of this study was to use Photovoice, a participatory research method, to explore the experiences and perceptions of communities in India on the role of dairy products in local sustainable and healthy food systems.

**Methods:**

Purposive sampling recruited two women’s self-help groups in Visakhapatnam, Andhra Pradesh: one in a rural area and one in an urban area. A total of 31 participants (10–17 urban group and 12–14 rural group), produced photographs with captions to represent their views on how dairy was produced, sold, and consumed in their community. A discussion workshop was held in each area, with prompts to consider health and the environment. Workshop transcripts, photographs and captions were analysed qualitatively using thematic analysis.

**Results:**

A range of experiences and perceptions were discussed by the two women’s self help groups. Participants had an awareness of their local food system and how stages of dairy food supply chains were non-linear and inherently interconnected. Three main themes were identified: 1) Quality and value matters to producers and consumers; 2) The need to adapt to sustain dairy farmer livelihoods in water scarce areas; 3) It’s not only about health.

**Conclusions:**

Moderate milk-producing states such as Andhra Pradesh will continue to develop their dairy industry through policy actions. Including communities in policy discussions through innovative methods like Photovoice can help to maximise the positive and minimise the negative role of dairy in evolving local food systems.

## Background

Community dialogues are increasingly used to facilitate complex discussions, such as how to adapt local food systems to work towards and beyond the 2030 UN Sustainable Development Goals (SDG) [1, 2]. Community-based engagement has many advantages as i) individuals can engage with issues to deepen their understanding; ii) people who do not usually have a say in the decisions that affect their daily lives are provided a platform; iii) communities can contribute to understanding complex societal issues; and iv) governments can have the opportunity to respond to the hopes and concerns of local communities [3, 4].

Managing the role of dairy in local food systems is a complex challenge for governments around the world. This is especially relevant in India, which is the largest milk producing nation in the world and will account for more than half the growth in global milk production over the next twenty years [5, 6]. Consumption of milk and milk products is also expected to increase, as income per capita rises throughout India. The challenge is deciding whether to support production/consumption demands and which routes to take to meet these demands, whilst considering the potential consequences of these actions in a holistic manner, including un/intended consequences for the economy, livelihoods, food safety, nutrition, environmental degradation, and equity throughout society as a whole.

Central and state governments in India have facilitated recent growth in dairy production. The 1960s Operation Flood (assisted by the World Food Programme) supported a co-operative dairy structure in India and initiated what has become known as a “White Revolution” [6]. Subsequent policies (Milk and Milk Product Orders in 1992 and 2002, and the 2020 Animal Husbandry Infrastructure Development Fund subsidy scheme), have all encouraged further modernisation and incentivised investment in the dairy sector [7].

A growing Indian dairy sector has the potential to improve livelihoods for dairy farmers and other actors throughout the milk supply chain (e.g., from production, through to processing, distribution and retail) [8]. The majority of milk in India is produced via a network of smallholder mixed farms (one to three cows), and approximately seventy-five million rural families in India are engaged in dairying activities [9]. This milk is sold via informal and formal markets: direct from farm gate, via third-party sellers, co-operative/private dairies, and stores [10]. Dairying has become an important secondary source of income for households engaged in livestock and agriculture, and the government views growth in the dairy industry as one route to fulfilling  a pledge to double farmers’ incomes, as well as contribute to national GDP [11].

Milk consumption is also associated with health benefits. Milk intake can be a proxy for good overall nutrition, and dairy products provide access to nutrients (e.g., calcium in milk), which promote the growth and development of young children [12]. Most of the milk produced in India is consumed domestically as either milk or milk products (butter/clarified butter/ghee, yoghurt/curd, cheese/paneer, flavoured milk and ice cream etc.). Consumption patterns are not universal across the country and can vary by region or community due to the level of deprivation/food security or in line with traditional practices [13]. Higher intakes are more likely to be observed in urban areas, wealthier households, and large milk producing states, such as Gujarat [14, 15].

The environmental impact of different models of dairy production and consumption has received increasing attention across the world. In high-income countries livestock are associated with high greenhouse gas equivalent emissions due to land cleared for pasture/feed, the resources required to grow livestock feed, and methane released over the lifetime of livestock [16]. Environmental impacts vary according to dairy practices. Higher yielding industrial dairy practices followed in high-income countries might require less land, yet have a higher environmental impact due to importing large amounts of livestock feed grown using land and water resources elsewhere [17]. In contrast, the lower yields from smallholder dairy farming in low- and middle-income countries, such as India, can require fewer resource inputs, yet overall a greater number of animals will be needed to produce the same amount of milk [18]. Furthermore, the degree of environmental impact will be dependent upon the context of the farming region, i.e. the vulnerability of a region to, for example, drought, as well as the extent of chemical pollution (antibiotic, pesticide, fertiliser use), which in turn can impact soil and water resources and damage bio-diversity [10, 16].

The withdrawal of WHO support from the EAT-Lancet planetary health diet highlights the tensions and challenges of balancing health, environmental sustainability, and equity (including livelihoods) priorities in different regions of the world [19]. The planetary health diet had hoped to discourage the over-consumption of resource intensive foods globally. The WHO however, did not believe enough consideration had been made for individual country contexts and the consequences for farmers or livelihoods dependent upon resource intensive foods, such as animal sourced products. The current study used the visual participatory research method of Photovoice to engage and promote critical dialogue in local communities through the use of photography, with the aim to explore community experiences and perceptions on the role of dairy products in local sustainable and healthy food systems.

## Methods

The study design was qualitative and used the visual participatory research method Photovoice (3). Methods have been reported in line with the Consolidated criteria for reporting qualitative research (COREQ) checklist [20].

### Setting

The study was set in the Visakhapatnam district in Andhra Pradesh, India, during 2019–2020. This district was conveniently sampled as the communities in this region were well known to the research team (see research team section below). Two local areas were purposively sampled within Visakhapatnam to represent one urban and one rural area, of similar low-middle economic status, and capture the experiences and perceptions of people who produce, purchase, and consume milk and milk products.

### Context

This study was part of a Sustainable and Healthy Food Systems (SHEFS) programme of research. The aim of SHEFS is to bring together inter-disciplinary research evidence and explore how future food system policies can deliver nutritious and healthy foods in a sustainable and equitable manner.

### Research team

The research team included SHEFS researchers and local health workers from the Public Health Foundation of India (PHFI). The research team were well known and had a good rapport with the local communities from implementing a diabetes and hypertension prevention and management programme (UDAY), in a population of ~ 200,000, in this area since 2013 [21]. There was already an open and trusted environment to facilitate engagement, and this made the current study design feasible.

### Recruitment

Women were purposively approached to take part in the study via women’s self-help-groups (SHGs). Women play an important role in both dairy production, purchase, and consumption practices across India, and SHGs are local voluntary women’s groups involved in a range of economic, social and community leadership initiatives across India [22]. They typically have 8–12 members and engage in income generating activities and/or can be a focal point for achieving community objectives [23]. These groups are used to coming together and discussing community issues, making the study design feasible and appropriate for this sample.

PHFI health workers met with community leaders and gained permission to conduct Photovoice workshops in their community. They then met community SHG members to discuss the proposed work and gain permission to recruit via two purposively sampled local SHGs (one rural and one urban). Information sessions were held in both local SHGs. Members were able to ask questions before deciding if they felt sufficiently informed to provide written consent and take part in the workshops. All members who attended these sessions agreed to participate (100% response rate).

### Participants

A total number of 31 participants were recruited over the study period. The number of participants in the workshops varied from 10–17 in urban Visakhapatnam and 12–14 in rural Visakhapatnam. All participants were female. The mean age of the participants was 42 years (range 32 to 49) in urban Visakhapatnam and 28 years (range 21 to 42) in rural Visakhapatnam. Groups were representative of women in the areas sampled, with similar socio-economic status, education, culture, and language backgrounds, which facilitated discussions and minimised barriers to participation.

### Ethics

Ethical approval was obtained from the London School of Hygiene & Tropical Medicine Ethics Committee, UK in Sep 2018 (LSHTM ethics reference: 14,664) and the institutional ethics committee of the Centre for Chronic Disease Control, India in March 2019 (CCDC reference IEC_02_2019). Participants provided written informed consent to take part in the study. In addition, written informed consent was obtained from all participants for specific images and data to be used in an open-access publication.

### Photovoice protocol

The Photovoice protocol was piloted with PHFI health workers to ensure the framing of the sessions and activities was appropriate for the local community. During the pilot, we realised that the concept of healthy and sustainable food systems was abstract and difficult to understand. To aid communication, in the first Photovoice session, the research team introduced a sustainable diets top-trumps/flash card game, where cards with information on nutritional (carbohydrates, proteins and fats) and environmental (carbon and water footprint) aspects of commonly consumed food items in India were listed [24].

Participants decided the time and date of the workshops and local PHFI health workers assisted the research team (NSV and GP) to run the Photovoice sessions. The materials and sessions were all available in the local language i.e., Telugu. Each group completed five workshops over the period of four weeks. The research team provided participants with easy-to-use digital point and shoot cameras and chargers to take home over the study period. SHG members were invited to learn basic photographic principles (e.g., concept of focus, subject, framing), and the ethics of taking and sharing photos. The main Photovoice workshops were used to discuss photographs taken by SHG members. Members were asked to take photographs to illustrate the role of dairy in their community. They were asked to consider different parts of the food supply chain (dairy production, retail, and consumption). These photographs were used to elicit discussions amongst SHG members. Discussions were led using the SHOWeD technique: a. What do you See? b. What is Happening? c. How does this relate to Our life? d. Why does this concern us? and e. What can we Do about it? [3]. Participants were prompted to reflect on both health and environmental issues. Participants selected their favourite photographs and captioned them to add contextual information including why the photograph was taken and what they wanted to convey through the picture and caption. Participants, PHFI health workers, and the research team used the last sessions to curate and hold photo exhibitions. This involved participants collectively selecting photographs and captions to design and then hold community photo exhibitions. These exhibitions were used to present the activities of the SHG members and discuss their views with the wider community (friends, family, neighbours, community leaders).

### Analysis

#### Data

Recordings were made of the main discussions of photographs taken by SHG members in one urban and one rural Photovoice workshop. Recordings were transcribed in Telugu. A total of 81 photographs and captions were produced for the two community photo exhibitions. The Telugu transcriptions together with the Telugu captioned photographs were translated into English to share analysis throughout the research team.

#### Thematic analysis

The aim of thematic analysis is to identify patterns/themes to understand a situation under study [25]. Thematic analysis is not necessarily attached to any particular theory and this allowed the diverse inter-disciplinary research team an accessible and flexible research tool [26]. A codebook or hybrid thematic analysis approach was taken [27]. This involved using an initial skeleton a priori coding template and following principles of reflexive qualitative research to identify and interpret themes e.g., becoming familiar with the data; iteratively generating codes and searching for themes; and reflecting upon and challenging interpretations of the data [28, 29].

First, a skeleton coding template was created a priori to explicitly reflect upon the study aim and guide initial analysis for the whole research team, including novice qualitative researchers. This template represented different areas of the food supply chain and mirrored the SHG Photovoice activities (dairy production, dairy retail, and dairy consumption), as well as the SHEFS project areas of interest (health and environment). Analysis was carried out using MS Word to enable data sharing between members of the research team. Second, the transcripts, photographs, and captions (in English) were initially coded by three researchers using the template. Once familiar with coding, codes were amended, sub-codes were identified, and new codes were added where necessary. There were few disagreements in categorisation and all three researchers identified similar codes. Third, two researchers (one English speaking, one Telugu speaking) looked across all the codes and the data set as a whole, to iteratively identify key themes and illustrative quotes (in English). This involved reflecting upon the aim of the study i.e., which topics participants particularly engaged with or promoted critical dialogue on the role of dairy products in local sustainable and healthy food systems community, as well as considering any surprising, or new findings. Key themes were discussed as a research group. This involved consultation with a fourth researcher, a Telugu speaker involved in the data collection, to check the authenticity of data analysis and representation.

## Results

Three main themes were identified: 1) Quality and value matters to producers and consumers; 2) The need to adapt to sustain dairy farmer livelihoods in water scarce areas; 3) It’s not only about health. Results are presented below by theme, using illustrative quotes from workshop transcripts, as well as displaying the photographs and captions, which elicited particular discussions (labelled urban or rural).

### Quality and value matters to producers and consumers

Quality and value were mentioned throughout the discussions, by both urban and rural participants, and in relation to all parts of the milk food supply chain (production, retail, and consumption). Urban participants, a minority of whom were involved in milk production, discussed quality as an end-user/consumer in terms of trust in the quality of milk produced. The rural participants, a large number of whom were involved in producing milk, discussed the value of producing a high quality milk product. Both groups discussed the value to local communities of dairy production as a traditional livelihood and dairy consumption as a traditional pastime.

#### Value of producing high quality milk

**Fig. 1:**
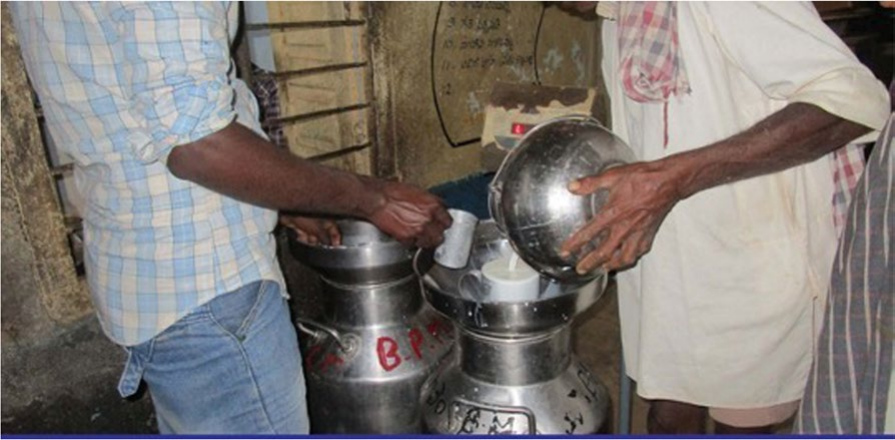
We buy milk from milk centres. Clean and unadulterated milk is available there which is good for health [SHG 2, rural]

Rural participants referred to the value of producing high quality milk to improve livelihoods i.e., aiming for a higher quality product, with a higher fat content, to achieve a higher price at milk collection centres and receive greater benefits to the wider community. Figure 1 refers to a low risk of adulteration or poor quality products at milk collection centres. This photograph elicited discussions about the value of high quality milk production and practices to increase milk quality, such as milking in the morning, good animal husbandry, and avoiding milking when animals are in ill-health. Value in terms of the greater number of milk products that could be made from a higher quality (high fat) milk product was also discussed.

“We get more money if the butterfat volume in the milk is more. We get less money if there is lesser butterfat volume.” [SHG 2, rural]

“Even in terms of quality, more ghee and butter come from the milk because it is extracted early in the morning. Lesser butter and ghee come from the milk extracted during nights.” [SHG 2, rural]

The value of being part of a co-operative dairy was discussed at length with rural participants, and how benefits were distributed dependent on the quality and quantity of milk sold to the dairy. Bonuses would be given throughout the year (e.g., at the *Sankranthi* harvest festival celebrated in January and prior to the start of the academic year). This was on top of access to free veterinary care (medicines to be paid in advance, veterinarian time and expertise provided by the dairy), family healthcare provision, health insurance for family members, interest free loans, and contributions to community facilities such as schools, producing wider community benefits “…for children, scholarships, and health care at community clinics…”.

“It depends on the milk we sell. Sir, you have certain deductions when you draw a salary, the same way they deduct a certain amount depending on how much milk we supply to them and that is given as bonus.” [SHG 2, rural]

#### Value of trusting the quality of milk purchased

**Fig. 2:**
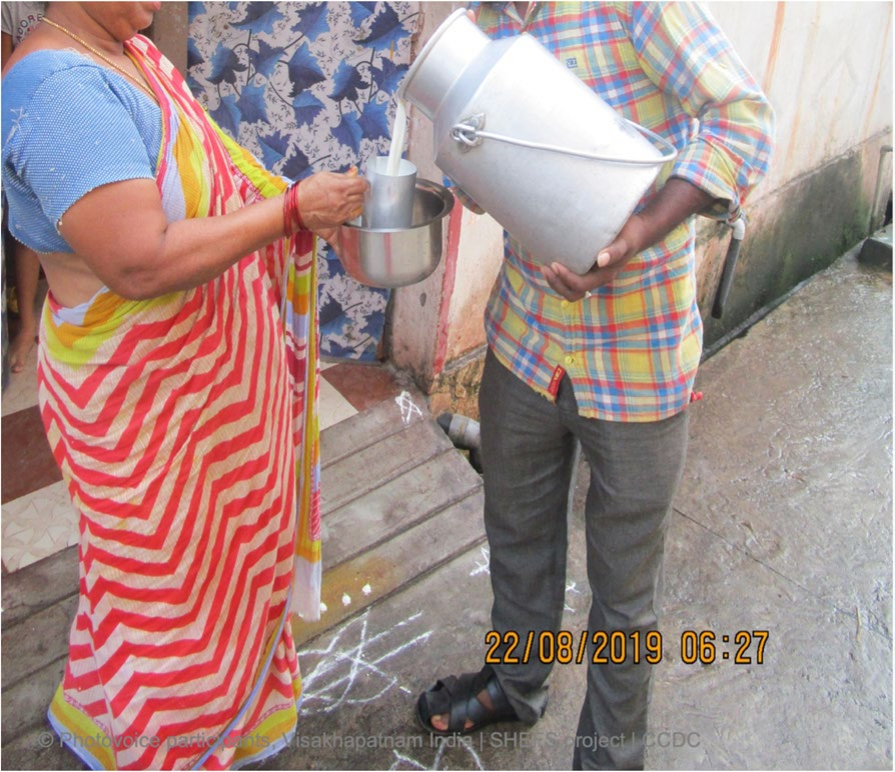
We are buying milk from him since childhood. We trust the milk that he supplies. So, I have clicked his picture [SHG 1, urban]

Trust was a key factor in judging the quality of milk purchased. A higher quality product was considered to be tastier and unadulterated due to its higher fat content i.e., not watered down. Figure 2 elicited a long discussion in the urban group on the adulteration of milk and a preference for purchasing fresh milk directly from a farmer at the time of milking. The photographer explained they purchased milk from a familiar farm, one they trusted, somewhere their family have bought milk since they were a child.

Other participants remarked that they would go out of their way (early in the morning or a distance) to collect milk from someone they trusted, where they could be confident water had not been added to the raw milk “that is the reason, we go even before they could extract the milk”. This was preferred over the convenience of having milk delivered to their home, as if “they deliver it to our home but then we would not be able to trust” and “once it changes hands, anything can happen”.

**Fig. 3:**
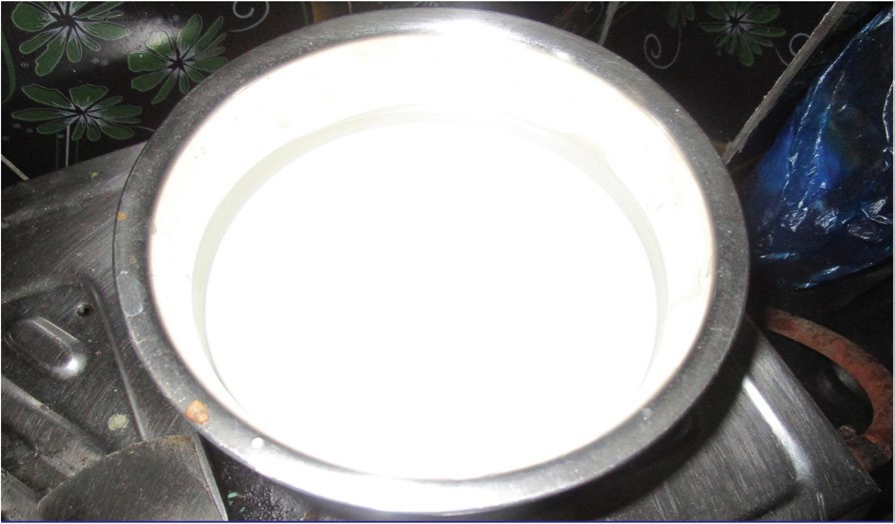
Milk should be boiled before consumption. This will ensure that harmful bacteria are killed. We also come to know if the milk is fresh or not [SHG 1, urban]

Participants generally preferred to receive ‘trusted’ raw (cow/buffalo) milk or “fresh milk that comes daily”, over milk available in plastic packets or Tetra Paks. They were aware raw milk needed to be boiled before consumption to remove pathogens, whereas packaged milk had already been pasteurised or processed at ultra-high temperatures (UHT milk) and “stays fresh for a longer time”. Nevertheless, boiling milk was habitual and often used as a way to test whether a product was fresh (Fig. 3).

“You get vomiting if you drink raw milk. So we boil it and drink.” [SHG 2, urban]

“…[milk] from the cow curdles if we keep it at room temperature for a day. This [packet] milk does not spoil for a day even after opening”. [SHG 2, rural]

Tetra Paks were described as having several advantages, such as being convenient when away from home, where boiling on site would be difficult, and/or catering for large numbers as “they are useful when you go camping and such”. The process to preserve the milk, however, was thought to alter the taste and fresh raw milk was still preferred.

“…it is preserved for a long period, right and it is not as tasty.” [SHG 1, urban].

All participants discussed the preparation of milk products and milk dishes at home (e.g., yoghurt/curd, buttermilk, and clarified butter/ghee; Payasam/Semya/khova, tea and coffee), as well as situations when they would prefer to purchase milk products from stores or dairies (e.g., milk sweets such as paneer jalebis, as well as ice-cream, lassi, and chocolate).

Products made at home were considered more hygienic, although store-bought products were necessary for large amounts of food at festivities, family occasions, or celebrations. There was also reference to the greater choice of stores/products available to urban participants, the greater availability of raw milk products to rural participants, and/or the cooking skills/knowledge of particular participants, which would inform whether products were made at home or purchased.

“When we make it at home, we make it on ourselves, it is hygienic. We make it with our very hands we know what we are using what we are not using.” [SHG 2, rural]

“These days, there is nothing that is not available outside. You get everything.” [SHG 1, urban]

“If they have in surplus, they sell it, sir. If they need it, large quantities for a party or for a wedding, they buy it from others.” [SHG 2, rural]

Urban participants preferred to buy certain products directly from the dairy, whilst others could be bought in local small stores (*Kirana*) or larger mini supermarkets. When buying in a store, participants mentioned brand preferences and a common practice of testing milk product samples, to check the freshness, before purchasing.

“The brand X is good. We also look at the expiry date. We look at the manufacturing date and the expiry date.” [SHG 1, urban]

“Anything [sweets made out of milk] we buy, we test it, and then we buy.” [SHG 1, urban]

### The need to adapt to sustain dairy farmer livelihoods in water scarce areas

**Fig. 4:**
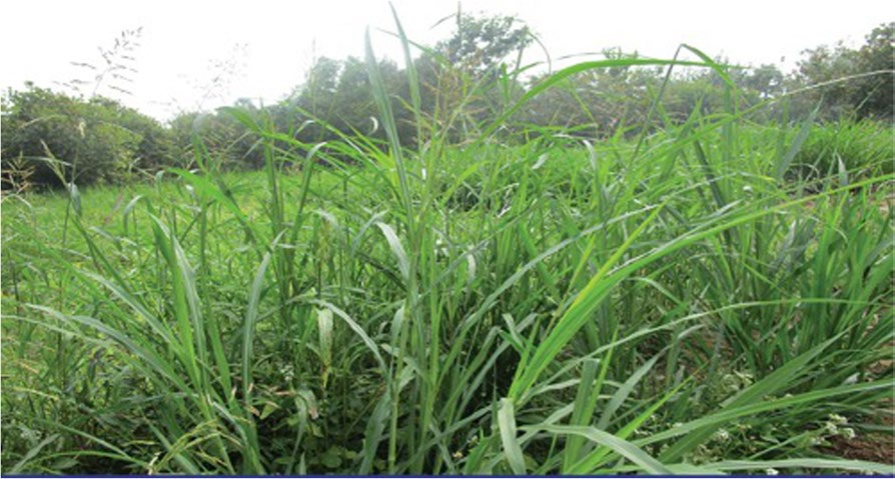
Green grass is the food for animals. Grow trees to keep environment clean and protect health. Greenery is because of the farmer. Food is available only because of the farmer [SHG 2, rural]

Concerns regarding dairy production livelihoods were identified; however, these were subtle, and related to promoting dairying as a viable livelihood and adapting to changes in the environment, predominantly due to water scarcity or irregular rains. Two photographs in the rural group represented the benefits to the community of a green and natural environment. One of these (Fig. 4) led to a substantial discussion on feed for cows and buffalos and how this has changed over the years to adapt to the lack of water in the region.

Participants mentioned the use of different feed (boiled broken rice and rice husk powder, peels and husk of pulses, hay) used to supplement the grass either fed to or grazed by cows/buffalos. They discussed the convenience of using a newer type of dairy grass and how this has led to a new generation of dairy farmers. This grass was described as beneficial as it required less water, grew quickly, and was less labour intensive. It was considered more economic than “natural grass” and part of why the dairy industry was becoming a more popular livelihood.

“The [natural] grass also does not grow because there are no rains.” [SHG 2, rural].

“Earlier there was no dairy grass. Now there is no water, so that grass is not growing. This grows if you just water the soil. That grass does not grow. So, we are used to using this. For it to grow soon urea is used. It grows in 15 days. It is easy for us, so we are cutting it and we feed it.” [SHG 2, rural]

“Earlier this grass was not there so no one was into farming. But now even people who are [not from traditional farming communities] can do it with this grass. As a result, cows are also more in numbers.” [SHG 2, rural]

The only explicitly negative association with dairy grass was the reaction to using urea, a fertiliser high in synthetic nitrogen. Urea was seen as a means to an end, and a common practice necessary due to the lack of water in the region; however, participants were uncomfortable with using chemicals and believed they would impact their health either directly or via the milk produced.

“The cattle are affected and in turn the milk coming from them is also affected.” [SHG 2, rural]

### It’s not only about health

**Fig. 5:**
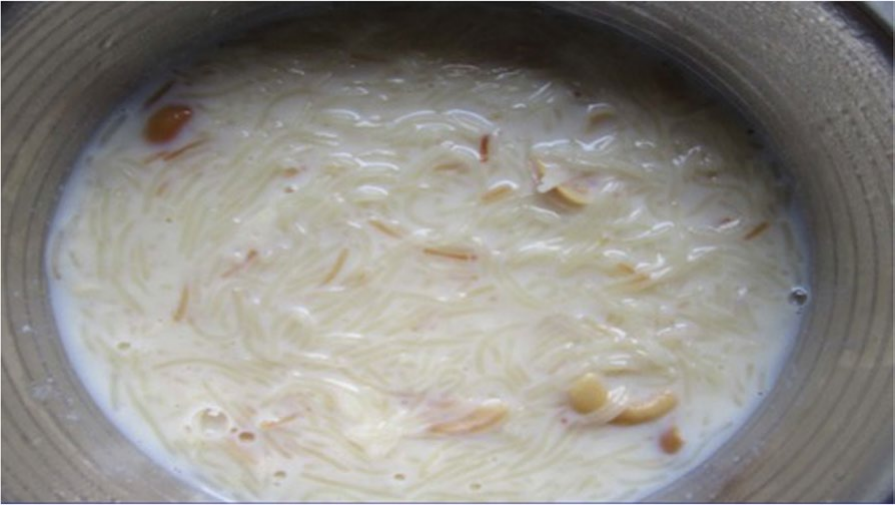
*Payasam* (vermicelli and milk dessert) is prepared on birthdays and festivals. All my family members like it [SHG 2, rural]

Participants in both the urban and rural groups referred to the enjoyment in cultural practices of offering and consuming milk and milk products. This was discussed as part of everyday customs as “It is customary to serve tea or coffee (with milk added) to the guests.”; as well as for family celebrations where “Sweets made out of milk are required to be served in functions. It is good if these sweets are served to the guests.”; and also for many religious festivals (Fig. 5).

“Semiya payasam is offered to God. It is also liked by my children.” [SHG 1, urban].

**Fig. 6:**
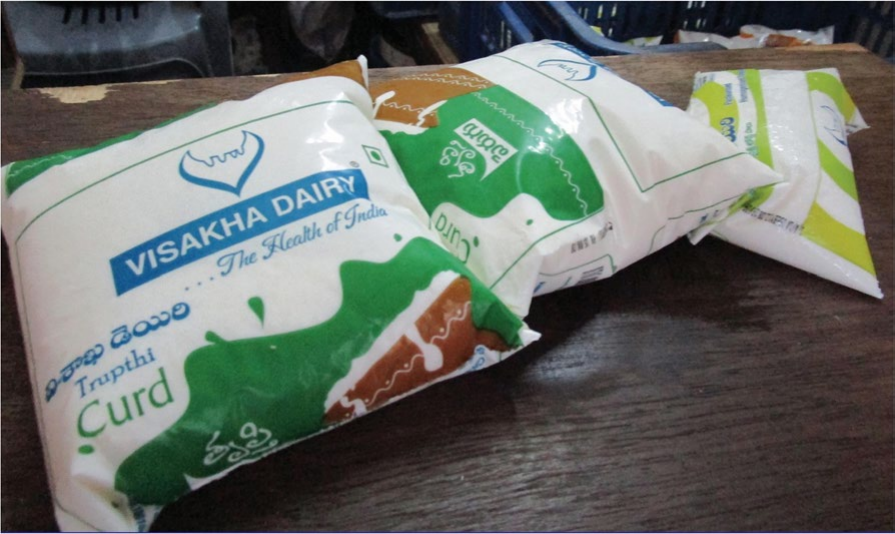
This is healthy for all and makes bones strong [SHG 1, urban]

**Fig. 7:**
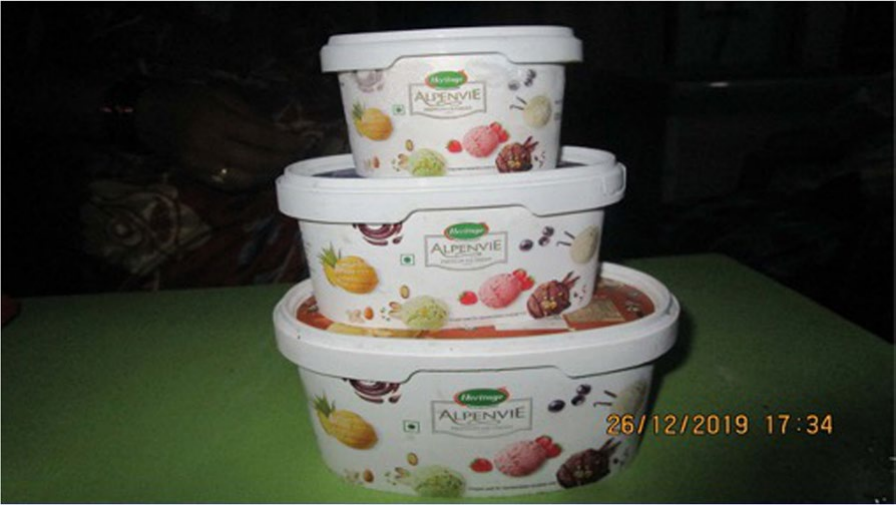
Ice cream is liked by children and adults alike. But too much ice cream is not good for health [SHG 2, rural]

The health benefits of consuming milk were clearly discussed amongst all participants, in particular for the health of children because “milk has calcium” and “it strengthens bones, too” (Fig. 6). Many participants, however, stated that health was not always the main reason for consuming milk and milk products. They spoke of preferences and liking the taste of a number of milk-based foods and drinks (e.g., ice cream, ghee, tea), whilst also recognising that should be consumed in moderation for health reasons (e.g., to moderate energy/fat intake) (Fig. 7).

“Chocolate, sir. It is made from milk. Children like it. Not only children but also adults enjoy it.” [SHG 1, urban]

## Discussion

This study represents the experiences and perceptions of female dairy consumers and producers in urban and rural Visakhapatnam, Andhra Pradesh. The SHG members were able to use the Photovoice workshops as a way to engage with their local food systems and communicate the positive and negative impacts of dairying on public health and the environment in their area. The research team also learnt the insights below from the communities involved.

First, the concept of quality milk supply chains described was wide ranging. SHG members were inherently aware of how elements of the food supply chain were linked and the universal benefits of a high quality system, where producers receive fair prices and consumers receive value for money in trusted, unadulterated, and nutritious products. The cooperative model of dairying was also valued. This aligns in part with government interventions aimed at improving the quality standards of dairy in India. Surveys by the FSSAI reported 90% of milk in India is safe to drink, and the frequent practice of boiling milk before consumption relayed in this study indicates the risk of ill-health from pathogens is unlikely to be widespread [30]. Nevertheless, there are still reports of adulteration, microbiological and heavy metal contamination, and overuse of antibiotics [10, 31, 32]. The FSSAI has introduced (voluntary) monitoring and surveillance hygiene guidelines and the Government of India has policy programmes to strengthen cold storage and efficiency in the food processing sector, as well as in the production of value-added milk products [33, 34]. These initiatives are primarily designed to capitalise on dairy commodity prices, especially in the international market where marginal milk and dairy exports originate from India and there are opportunities for trade, when international food standards can be met [5]. Future research can explore the impact of further liberalisation of the dairy industry on the domestic market and the co-operative model, which to date has been considered a successful way to protect smallholder livelihoods via its network of small mixed farming, local milk collection centres, and co-operative dairies [8, 35].

Second, dairy farmers are already adapting to climate change and variability (predominantly due to drought or irregular rains), and require accessible and attractive environmentally sustainable alternatives for year-round fodder. The rural community involved in this study was optimistic for a new generation of dairy farmers. Changes in farmer practices such as the use of ‘quick growing’ and ‘easy to crop’ grass for cattle fodder, as well as incentives to increase farmer incomes were considered to be positive in encouraging new generations into dairying. These came at a cost however, and there was concern over the sustainability of farming practices, in particular the water supplies available to grow the grass needed for cattle fodder. These findings reflect a nationwide challenge with meeting increased production demands, whilst using sustainable farming practices to protect farming livelihoods and public health, as well as the environment. India is susceptible to water stress and irregular rains in certain areas, and this is considered to be a substantial challenge to the future of agriculture and livestock farming [36]. Andhra Pradesh, in particular, has been identified as a resource poor state regarding both water and livestock fodder [37]. The use of ground water irrigation practices to grow grass for livestock fodder is associated with higher dairy yields, due to the higher quality and quantity of livestock fodder produced [38]; yet, if growth in the dairy industry were to result in switching from rainfed to ground water irrigation farming practices this could contribute to the overuse of and overreliance on groundwater, depleting aquifers and ultimately leading to competition between agricultural and drinking water supplies [36]. Ongoing engagement with rural and dairy production communities can help to understand the evolution of local dairy food systems, and assess the impacts of any changes in farmer practices in reaction to government interventions or environmental constraints.

Third, participants emphasised the enjoyable aspects of consuming milk and milk products and how they will always have a place in certain communities due to their cultural, religious, and traditional significance. The majority of research focuses on how food production and retail environments shape consumption practices, whereas this study supports those who increasingly recognise the role of culture and food traditions in shaping food systems [39–41]. Food cultures are complex and they evolve [40]. The authors of this study would have liked to have gone further to explore the meaning of sustainability in the communities sampled, as well as the degree to which global shifts to the commodification of milk and consumption of milk products have impacted them regarding any tensions in subsistence farming practices (e.g., the selling of milk prioritised over self-consumption) or changes in culturally appropriate diets (e.g., consumption of out-of-home processed milk products or homogenisation of diets).

The dairy sector will continue grow in India, to meet production, retail, and consumption demands, such as those discussed in this study, especially in the moderate dairy producing state of Andhra Pradesh [37]. This sector is also seen as a means through which women are empowered. The government of Andhra Pradesh has introduced Animal Induction Policy for Women Beneficiaries in 2020 to support women and develop the livestock sector in the state. It provides money transfers to women farmers to purchase cattle and buffaloes [42]. Previous research has suggested co-creation, involving local communities in local and national policy development and implementation, can be innovative and provide real-time feedback on policy plans, as well as increase transparency of the values and politics underlying interventions and programmes [43, 44]. Any policy or intervention targeting one part of the dairy food supply chain (e.g., dairy production or consumption practices) would benefit from in-depth engagement with communities, such as those sampled, to take account of local concerns and develop inclusive and feasible pathways to move towards more sustainable and healthier food systems.

### *Limitations *and* strengths*

The findings presented related to two SHGs, in one rural and one urban area of Visakhapatnam. This impacts the empirical transferability of our findings and results should be interpreted with caution when applied to different contexts or populations [45]. This study did not seek to etablish a random or representative sample drawn from a population. We were interested in exploring, in -depth, the specific perspectives of women who lived in rural and urban areas of Visakhapatnam. Future research would benefit from sampling peri-urban areas to explore the perspectives of these communities, which also play an important role in local dairy food systems [46]. Alternatively, it would be valuable to purposively sample low, medium, and high milk production/consumption states to gain perspectives from these different communities. The framing of the research project, which invited participants to focus on milk and milk products, different parts of the food supply chain, and prompted for reflection on health and environmental impacts also limited the range of stories and perspectives identified in this study.

The research team were unable to involve participants in the final analysis and the development of this manuscript, due to disruption from the COVID-19 pandemic. Care was taken for participants to select and caption their own photographs, and be fully involved in the curation of the Photovoice exhibitions in each area. The research team also frequently consulted with local Telugu speakers, involved in data collection, to consider alternative representations of the findings and check the authenticity of data analysis/interpretation. The COREQ checklist has also been used to transparently disclose the scientific context and purpose of this study, as well as the analysis approach taken, to aid reader interpretation. Nevertheless, this study would have benefited from testimonial validity (participant feedback), which is a common validation strategy for certain types of qualitative research [47].

This work sought to his work has sought to represent the views of the local community that participated, providing a rich account of their experiences. Photovoice has been used previously to explore food security in climate sensitive populations, and regarding populations and changes to food environments [48], yet, to our knowledge this is the first time the role of an individual food group has been used to follow or ‘trace’ the impact of milk through the food supply chain and explore complex interactions with health and the environment. This work has used vivid local stories to illustrate the delicate balance between benefits and concerns for health, the environment, and society (equity/livelihoods and cultural practices). This would not be possible using traditional research methods, which invariably use one disciplinary approach and rarely capture such a range of in-depth views [49]. The United Nations Food Systems Summit 2021 has actively encouraged the development of community dialogues to explore and adapt complex local food systems to achieve and move beyond the UN SDG, although there have been criticisms of these dialogues regarding corporate influence [1, 48, 50]. The current study provides a different perspective on community engagement and makes a contribution to ongoing conversations on how to adapt our food systems, so that they are healthy, environmentally sustainable, and respond to the hopes and concerns of local communities.

## Conclusion

Moderate milk-producing states such as Andhra Pradesh will continue to develop their dairy industry through policy actions. Including communities in policy discussions through innovative methods like Photovoice can help to maximise the positive and minimise the negative role of dairy in evolving local food systems.

## Data Availability

The data are available from the corresponding author on reasonable request.

## Abbreviations

CCDC: Centre for Chronic Disease Control
COREQ: Consolidated Criteria for Reporting Qualitative Research
GDP: Gross Domestic Product
LSHTM: London School of Hygiene & Tropical Medicine
PHFI: Public Health Foundation of India
SDG: Sustainable Development Goals
SHEFS: Sustainable and Healthy Food Systems
SHG: Women’s Self Help Group
UN: United Nations
